# Food poverty among children aged 6–59 months in Brazil: results from the Brazilian National Survey on Child Nutrition (ENANI-2019)

**DOI:** 10.1017/S1368980024001435

**Published:** 2024-09-13

**Authors:** Letícia Barroso Vertulli Carneiro, Elisa Maria de Aquino Lacerda, Natália Oliveira, Raquel Machado Schincaglia, Nadya Helena Alves-Santos, Talita Lelis Berti, Sandra Patricia Crispim, Dayana Rodrigues Farias, Juliana Vieira de Castro Mello, Paula Normando, Inês Rugani Ribeiro Castro, Gilberto Kac

**Affiliations:** 1 Instituto de Estudos em Saúde Coletiva, Universidade Federal do Rio de Janeiro, Rio de Janeiro, Brasil; 2 Departamento de Nutrição e Dietética, Instituto de Nutrição Josué de Castro, Universidade Federal do Rio de Janeiro, Rio de Janeiro, Brasil; 3 Departamento de Nutrição Social e Aplicada, Instituto de Nutrição Josué de Castro, Universidade Federal do Rio de Janeiro, Rio de Janeiro, Brasil; 4 Faculdade de Nutrição, Universidade Federal de Goiás, Goiânia, Brasil; 5 Instituto de Ciências da Saúde, Universidade Federal do Pará, Belém, Brasil; 6 Departamento de Nutrição, Universidade Federal do Paraná, Curitiba, Brasil; 7 Instituto de Nutrição, Universidade do Estado do Rio de Janeiro, Rio de Janeiro, Brasil

**Keywords:** Dietary diversity, Food poverty, Child, Survey

## Abstract

**Objective:**

To describe the prevalence of food poverty according to dimensions of socio-economic inequality and the food groups consumed by Brazilian children.

**Design:**

Dietary data from a structured qualitative questionnaire collected by the Brazilian National Survey on Child Nutrition (ENANI-2019) were used. The new UNICEF indicator classified children who consumed 3–4 and <3 out of the eight food groups as living in moderate and severe food poverty, respectively. The prevalence of consumption of each food group and ultra-processed foods (UPF) was estimated by level of food poverty according to age categories (6–23; 24–59 months). The most frequent combinations of food groups consumed by children living in severe food poverty were calculated. Prevalence of levels of food poverty were explored according to socio-economic variables.

**Setting:**

123 municipalities of the five Brazilian macro-regions.

**Participants:**

12 582 children aged 6–59 months.

**Results:**

The prevalence of moderate and severe food poverty was 32·5 % (95 % CI 30·1, 34·9) and 6·0 % (95 % CI 5·0, 6·9), respectively. Children whose mother/caregiver had lower education (<8 years) and income levels (per capita minimum wage <¼) had the highest severe food poverty prevalence of 8·3 % (95 % CI 6·2, 10·4) and 7·5 % (95 % CI 5·6, 9·4), respectively. The most consumed food groups among children living in food poverty in all age categories were ‘dairy products’, ‘grains, roots, tubers, and plantains’ and ‘ultra-processed foods’.

**Conclusion:**

Food poverty prevalence was high among Brazilian children. A significant occurrence of milk consumption associated with grains and a considerable prevalence of UPF consumption were found among those living in severe food poverty.

Adequate nutrition promotes optimal health, growth and development during infancy and early childhood. Complementary feeding, which generally occurs between 6 and 24 months of age, should include a variety of food groups to ensure that nutrient requirements are met^(1)^. The Brazilian Dietary Guidelines^(2,3)^ recommend that diet be diverse, varied and based on unprocessed or minimally processed foods. They also recommend that ultra-processed foods (UPF) should not be offered to children under two years of age and should be avoided among the older ones to promote their optimal health, growth and development^(3)^.

The Minimum Dietary Diversity (MDD) is one of the core indicators for infant and young child feeding practices. It is defined as the percentage of children aged 6–23 months who consumed foods and beverages from at least five of the following eight food groups: (1) breast milk; (2) grains, roots, and tubers; (3) legumes; (4) dairy products; (5) flesh foods; (6) eggs; (7) vitamin A-rich fruits and vegetables and (8) other fruits and vegetables during the previous day^(1)^. Consuming a varied and diverse diet is strongly linked to the consumption of sufficient nutrients among children aged < 5 years^(4)^. Not achieving MDD may impair normal physical growth, brain development, and cognitive development, affect immunity, and increase the risk of infections and mortality^(5)^. Several studies among children aged < 5 years found an inverse association between MDD and being stunted, wasted and underweight^(6–8)^. Inadequate energy and nutrient intake in this age group affect educational performance and lifelong job opportunities and increase the risk of health problems in adult life^(5,9)^. Furthermore, the lack of MDD is influenced by various factors, including younger child age, mothers with lower age and education, inadequate number of prenatal visits (less than five), limited access to healthcare services^(10)^, and mainly the unavailability or inaccessibility of food^(11)^.

Food poverty is a new indicator proposed by UNICEF^(12)^ and represents the lack of MDD. Children who consumed 3–4 and < 3 of the eight food groups are classified as living in moderate and severe food poverty, respectively.

Globally, 41 % and 30 %, and in Latin America and the Caribbean, 28 % and 10 % of children live in moderate and severe food poverty, respectively. Moreover, children in poor and rural households are more vulnerable to severe food poverty. In low-income (35 %) and lower-middle-income (31 %) countries, the prevalence of children living in food poverty is three times higher than that of upper-middle-income countries (11 %). Only 10 % of children in severe food poverty receive food other than breast milk/dairy products and grains, roots, and tubers. It is staggering that out of the 202 million children aged < 5 years who live in severe food poverty globally, 41 % consume less than two of the eight recommended food groups^(12)^.

Although the MDD indicator has been extensively studied, there is a lack of data regarding food poverty among children in Brazil, and the recently published UNICEF report^(12)^ is currently the unique global source. Therefore, this study aims to describe the prevalence of moderate and severe food poverty, examine three dimensions of socio-economic inequality (maternal level of education, family income and food insecurity level) and explore variations according to geographic region in a representative sample of Brazilian children aged 6–59 months. The study also aims to describe the food group combinations consumed by children living in severe food poverty and the consumption of UPF in children experiencing some level of food poverty.

## Methods

### Study design, sampling and population

The data are from the Brazilian National Survey on Child Nutrition (ENANI-2019), a national household survey with a complex probabilistic sample that examined 14 558 Brazilian children aged < 5 years^(13,14)^. The sample design of ENANI-2019 used stratification and clustering, incorporating two or three selection stages. The primary sampling units were the municipalities or census enumeration areas, and the elementary sampling units were the households with at least one child aged < 5 years on the survey interview date. The ENANI-2019 sample is representative of Brazil’s five geographic regions (North, Northeast, Southeast, South and Midwest), children’s age groups (6–23 and 24–59 months) and sex. More details related to the questionnaire and data collection have been published previously^(13)^. The 12 582 children aged 6–59 months surveyed in the ENANI-2019 were included in the present study. Children < 6 months were outside the complementary feeding age range; therefore, they were excluded.

### Study variables

The questionnaire used in ENANI-2019 included forty items regarding markers of the consumption of food groups on the day before the interview. Questions were designed to allow the evaluation of the (i) WHO indicators for feeding practices in children aged < 2 years^(1)^ and (ii) Brazilian Ministry of Health indicators. The food groups comprise (1) breast milk, (2) grains, roots and tubers such as porridge, bread, rice, pasta, potatoes and other starchy vegetables, (3) legumes including beans, lentils, peas and chickpeas; (4) milk and dairy products like infant formula, animal milk and yogurt; (5) flesh foods that contain animal meat, liver, kidney, heart, sausages and processed meats; (6) eggs; (7) vitamin A-rich fruits and vegetables such as carrot, pumpkin, sweet potato, cabbage, spinach and other local dark green leafy vegetables and (8) other fruits and vegetables.

Items about UPF consumption^(15)^ were also included in the questionnaire: sweet or savoury biscuits/cookies; baby cereals; yogurts; carbonated drinks; other sweetened beverages (packed juice, packed coconut water, guarana syrups, redcurrant syrup, powdered juice or fruit juice with added sugar); sweets and treats; sausages and processed meats; and packaged snacks, packaged bread and instant noodles^(16)^. An overlap of foods from the UPF groups with three of the eight groups used to evaluate food diversity may have occurred: ‘grains, roots, tubers, and plantains’; ‘dairy products’ and ‘flesh foods’. This happened because UPF, such as baby cereals, yogurt and processed meats, belong to these groups.

Food poverty was defined as the percentage of children aged < 5 years consuming foods and beverages from four or fewer of the eight food groups. Moderate and severe food poverty occur if the child consumes 3–4 groups and two or fewer groups, respectively^(12)^.

The dietary profile of children living in severe food poverty was examined to determine the combinations of food groups they consume.

Children who consumed only UPF were classified as ‘ultra-processed foods only’; if the child did not eat any food listed the day before the interview, they were classified as ‘none of the food groups listed’. The food poverty indicator did not include the UPF group (‘UPF only’).

Given the high prevalence of consumption of UPF among Brazilian children^(17)^, as well as the growing evidence on the impact of the consumption of UPF on the diet quality and health outcomes among children^(18,19)^, it was considered relevant to describe the consumption of these foods among children experiencing some level of food poverty. This fact could further worsen the nutritional status of these individuals, although the UNICEF indicator does not consider these foods as a specific group.

The socio-economic and demographic variables used in this study were the child’s age (6–23 and 24–59 months), Brazilian geographic regions (North, Northeast, Southeast, South and Midwest), educational level of the child’s mother/caregiver (0–7; 8–10; 11; ≥ 12 years of study), per capita family income categorised into minimum wage categories (R$ 998·00 (∼ US$ 252·88) in 2019 and R$ 1,039·00 (∼US$ 247·90) in 2020) (< ¼, ¼ –½, > ½–1, > 1), food insecurity, assessed using the Brazilian Food Insecurity Scale (EBIA) for households with residents < 18 years of age (food security; mild food insecurity, moderate food insecurity and severe food insecurity) and self-reported skin colour/race of the child’s mother/caregiver (white, brown and black). The EBIA is an adapted version of the US Department of Agriculture Food Insecurity Module with fourteen items. The scale was validated for the Brazilian population in 2004^(20)^ and referred to the three months preceding the interview^(21,22)^. Given the low representation of indigenous and yellow (Asian origin, such as Japanese, Chinese and Korean) self-reported skin colour/race of the mother/caregiver (<1 %), the estimates for these subgroups were not presented.

### Data analysis

The missing data were imputed for the variables of interest in this article using the automatic hot deck method, in which participants with similar socio-economic and demographic characteristics (geographic regions, sex age, and income quartile) donated data. This method is described elsewhere^(14)^.

The children’s socio-economic and demographic characteristics were calculated using frequencies and 95 % CI. The prevalence and 95 % CI of children living in moderate and severe food poverty were described according to age categories and geographic regions. The prevalence of consumption of the food groups included in the food poverty indicator and the UPF group was estimated for age categories and was also stratified by the level of food poverty (no food poverty, moderate and severe). For children living in severe food poverty, the most frequent combinations of food groups consumed were shown in figures according to age categories and geographic regions.

Equiplots were used to represent the distribution and disparities of the moderate and severe food poverty prevalence according to categories of the educational level of the mother/caregiver of the child, per capita family income categories and food insecurity level. These analyses were performed using STATA version 15.0, applying commands available on the International Center for Equity in Health from the Pelotas Federal University website (www.equidade.org/equiplot)^(23)^. Equiplot enables the visualisation of various indicators and prevalence among different groups, facilitating the analysis of health disparities. The wider the gap between the groups, the more pronounced the level of inequality.

A statistically significant difference was determined by the lack of overlap in the prevalence of 95 % CI between the groups. We calculated the estimates of the absolute number of children considering population totals and the CV of the estimates. The CV is a measure of dispersion that indicates data heterogeneity obtained by the ratio between the se and the estimated indicator value. We assumed that results with a CV ≤ 30 % had adequate precision, and CV > 30 % was interpreted cautiously. The analyses were performed with the R programming language^(24)^ using the functions of the *srvyr* and *survey* packages, considering the structure of the sampling plan, the weights and the calibration to compensate for non-responses and to match the population estimates with the total known population.

## Results

Most Brazilian children studied were > 24 months old (66·6 %), lived in the Southeast (39·2 %) or Northeast (28·1 %), and 48·6 % experienced some level of food insecurity (Table 1).

**Table 1:** Distribution of Brazilian children aged 6–59 months according to socio-economic and demographic characteristics. ENANI-2019

Variables	Frequency (%)	95 % CI
Age (months) 6–23	33·4	33·3, 33·5
24–59	66·6	66·5, 66·7
Brazilian geographic regions		
North	10·9	10·9, 10·9
Northeast	28·1	28·0, 28·2
Southeast	39·2	39·1, 39·3
South	13·5	13·4, 13·5
Midwest	8·3	8·3, 8·3
Maternal/caregiver formal education (completed years)		
0–7	22·8	20·9, 24·6
8–10	21·3	19·4, 23·2
11	39·1	36·9, 41·4
≥ 12	16·8	15·1, 18·5
Income (per capita minimum wage)*		
< ¼	29·1	26·0, 32·2
¼–½	33·3	31·0, 35·6
> ½–1	25·0	22·6, 27·4
> 1	12·6	11·0, 14·1
Food insecurity		
Severe food insecurity	4·2	3·2, 5·2
Moderate food insecurity	6·3	5·2, 7·4
Mild food insecurity	38·1	33·5, 42·6
Food security	51·4	46·3, 56·7

* Minimum wage for 2019 = R$ 998.00 (∼ US$ 252.88) and for 2020 = R$ 1,039.00 (∼ US$ 247.90).

Among children aged 6–59 months, 6·0 % lived in severe food poverty, being more prevalent in children aged 6–23 months (8·4 %, 95 % CI 6·8 %, 9·9 %) than in those aged 24–59 months (4·8 %, 95 % CI 3·6 %, 5·9 %). Children from the Northeast region of Brazil showed a significantly higher prevalence of severe food poverty when aged 6–59 months (8·9 %, 95 % CI 6·6, 11·3) and 6–23 months (13·3 %, 95 % CI 9·2, 17·4) compared with the Southeast region, which presented prevalence of 3·7 % (95 % CI 2·5, 4·9) and 5·6 % (95 % CI 3·4, 7·8), respectively (Table 2).

**Table 2:** Prevalence of food poverty in Brazilian children aged 6–59 months according to macro-regions. ENANI-2019

Macro-regions by age (months)	Severe food poverty		Moderate food poverty		No food poverty	
	%	95 % CI	%	95 % CI	%	95 % CI
6–59 months						
Brazil	6·0	5·0, 6·9	32·5	30·1, 34·9	61·5	58·7, 64·4
North	7·3	3·5, 11·0	39·4†,‡	34·5, 44·4	53·3†	46·2, 60·4
Northeast	8·9*	6·6, 11·3	37·9	33·0, 42·7	53·2	47·1, 59·2
Southeast	3·7*	2·5, 4·9	27·5†	22·9, 32·1	68·8†	63·6, 74·0
South	5·6	2·8, 8·3	29·7‡	26·1, 33·3	64·0	59·8, 69·7
Midwest	5·5	3·9, 7·1	32·8	28·4, 37·2	61·7	56·8, 66·7
6–23 months						
Brazil	8·4	6·8, 9·9	28·3	25·1, 31·4	63·3	59·9, 66·9
North	6·9	4·5, 9·3	38·3†	32·0, 44·6	54·8†	47·4, 62·1
Northeast	13·3*	9·2, 17·4	29·4	23·8, 35·1	57·3	50·5, 64·0
Southeast	5·6*	3·4, 7·8	25·1†	18·8, 31·4	69·3†	62·8, 76·0
South	7·9	4·2, 11·6	27·7	21·1, 34·4	64·4	56·5, 72·2
Midwest	7·4	5·1, 9·8	26·8	21·8, 31·8	65·8	60·4, 71·1
24–59 months						
Brazil	4·8	3·6, 5·9	34·6	31·9, 37·3	60·6	57·4, 64·0
North	7·4||	2·8, 12·1	40·0†	33·7, 46·3	52·6†	44·1, 60·9
Northeast	6·7	4·1, 9·4	42·2	36·4, 48·0	51·1*,§	43·9, 58·3
Southeast	2·8||	1·1, 4·5	28·8†	23·9, 33·6	68·4*,†	62·6, 74·3
South	4·4||	1·8, 7·1	30·6	26·0, 35·3	70·0§	59·0, 70·8
Midwest	4·5	2·8, 6·2	35·8	30·8, 40·7	59·7	54·3, 65·2

Note: severe food poverty: consumption of 0–2 food groups; moderate food poverty: consumption of 3–4 food groups; and no food poverty (i.e. with minimal dietary diversity): consumption of five or more of the eight food groups (i.e. 1) breast milk; 2) grains, roots and tubers such as porridge, bread, rice, pasta, potatoes and other starchy vegetables; 3) legumes including beans, lentils, peas and chickpeas; 4) milk and dairy products like infant formula, animal milk and yogurt; 5) flesh foods that contain animal meat, liver, kidney, heart, sausages and processed meats; 6) eggs; 7) vitamin A-rich fruits and vegetables such as carrot, pumpkin, sweet potato, cabbage, spinach and other local dark green leafy vegetables; and 8) other fruits and vegetables).

* Significant difference between Northeast and Southeast based on 95 % CI non-overlap.

† Significant difference between North and Southeast.

‡ Significant difference between North and South.

§ Significant difference between Northeast and South.

|| CV ≥ 30 %. CV is a measure of dispersion that indicates data heterogeneity, obtained by the ratio between the se and the estimated value of the indicator.

The prevalence of moderate food poverty was 32·5 % and was significantly higher in children aged 24–59 months (34·6 %; 95 % CI 31·9, 37·3) compared to those aged 6–23 months (28·3 %; 95 % CI 25·1, 31·4). The same pattern occurred for those living in the North compared with the Southeast region for all age categories and for children aged 6–59 months in the North (39·4, 95 % CI 34·5, 44·4) compared with the South (29·7, 95 % CI 26·1, 33·3) (Table 2).

Overall, the most consumed food groups by children were ‘Grains, roots, tubers, and plantains’, ‘UPF’, ‘Flesh foods’ and ‘Dairy products’ (Supplementary Table 1). Among children aged 6–59 months living in severe food poverty, the most consumed food groups were: ‘grains, roots, tubers, and plantains’ (49·9 % (95 % CI 43·0, 56·8)), ‘dairy products’ (49·9 % (95 % CI 42·1, 57·7)) and ‘flesh foods’ (23·8 % (95 % CI 17·9, 29·8)). In children living in moderate food poverty, the most consumed food groups were ‘grains, roots, tubers, and plantains’ (93·5 % (95 % CI 91·9, 95·2)), ‘dairy products’ (80·5 % (95 % CI 78·2, 82·8)) and ‘flesh foods’ (70·5 % (95 % CI 67·0, 74·0)). The frequency of children consuming UPF was higher in ‘no food poverty’ (children who achieved MDD) (92·2 % (95 % CI 90·7, 93·7)) and in moderate (86·9 % (95 % CI 84·4, 89·4)) than in severe food poverty (64·6 % (95 % CI 58·3, 70·9)) (Table 3).

**Table 3:** Relative frequency of food groups consumed by children aged 6–59 months according to categories of food poverty. ENANI-2019

Food groups by age	Severe food poverty		Moderate food poverty		No food poverty	
	%	95 % CI	%	95 % CI	%	95 % CI
6–59 months						
Breast milk	21·9	15·9, 27·8	16·6	14·0, 19·1	28·6	26·5, 30·6
Grains, roots, tubers and plantains	49·9	43·0, 56·8	93·5	91·9, 95·2	99·0	98·5, 99·5
Legumes	3·9	2·1, 5·7	54·7	50·4, 59·0	86·6	84·9, 88·4
Dairy products	49·9	42·1, 57·7	80·5	78·2, 82·8	90·8	89·2, 92·4
Flesh foods	23·8	17·9, 29·8	70·5	67·0, 74·0	93·0	92·1, 93·9
Eggs	3·0*	0·4, 5·6	9·9	7·6, 12·3	27·0	25·2, 28·8
Vitamin A-rich fruits and vegetables	1·1*	0·0, 2·2	8·4	6·9, 9·8	55·3	52·5, 58·0
Other fruits and vegetables	9·3	5·6, 13·0	31·1	27·8, 34·4	88·9	87·4, 90·5
Ultra-processed foods†	64·6	58·3, 70·9	86·9	84·4, 89·4	92·2	90·7, 93·7
6–23 months						
Breast milk	40·2	28·9, 51·4	46·6	41·4, 51·7	55·1	50·9, 59·3
Grains, roots, tubers and plantains	44·2	33·3, 55·0	86·3	82·1, 90·5	98·0	96·6, 99·3
Legumes	2·3*	0·7, 3·9	47·9	42·2, 53·7	80·5	77·1, 84·0
Dairy products	54·2	43·5, 64·9	78·9	74·0, 83·8	85·5	82·3, 88·7
Flesh foods	8·6*	1·3, 15·9	45·5	39·9, 51·0	89·6	87·7, 91·6
Eggs	1·1*	0·0, 3·3	5·5	2·6, 8·5	19·5	16·8, 22·2
Vitamin A-rich fruits and vegetables	0·2*	0·0, 0·5	14·6	11·1, 18·1	61·6	57·5, 65·7
Other fruits and vegetables	10·1	4·7, 15·6	39·0	34·2, 43·8	89·5	87·4, 91·5
Ultra-processed foods†	47·9	39·6, 56·1	76·1	71·3, 80·8	86·8	83·7, 89·9
24–59 months						
Breast milk	5·7*	1·4, 10·0	4·3	2·8, 5·8	14·7	12·3, 17·0
Grains, roots, tubers and plantains	54·9	46·5, 63·3	96·5	95·0, 98·0	99·5	99·2, 99·8
Legumes	5·3	2·3, 8·3	57·5	52·8, 62·2	89·8	88·3, 91·3
Dairy products	46·1	36·7, 55·5	81·1	78·7, 83·6	93·6	92·2, 95·1
Flesh foods	37·3	28·3, 46·3	80·8	77·4, 84·2	94·8	93·3, 96·2
Eggs	4·6*	0·8, 8·4	11·7	9·2, 14·3	30·9	28·5, 33·3
Vitamin A-rich fruits and vegetables	1·8*	0·0, 3·9	5·9	4·4, 7·3	51·9	49·0, 54·9
Other fruits and vegetables	8·6*	3·2, 14·1	27·8	23·8, 31·8	88·6	86·6, 90·6
Ultra-processed foods†	79·4	71·1, 87·8	91·3	89·1, 93·5	95·0	93·7, 96·4

Note: severe food poverty: consumption of 0–2 food groups; moderate food poverty: consumption of 3–4 food groups; and no food poverty (i.e. with minimal dietary diversity): consumption of five or more of the eight food groups (i.e. 1) breast milk; 2) grains, roots and tubers such as porridge, bread, rice, pasta, potatoes and other starchy vegetables; 3) legumes including beans, lentils, peas and chickpeas; 4) milk and dairy products like infant formula, animal milk and yogurt; 5) flesh foods that contain animal meat, liver, kidney, heart, sausages and processed meats; 6) eggs; 7) vitamin A-rich fruits and vegetables such as carrot, pumpkin, sweet potato, cabbage, spinach and other local dark green leafy vegetables; and 8) other fruits and vegetables).

* CV ≥ 30 %. CV is a measure of dispersion that indicates data heterogeneity, obtained by the ratio between the se and the estimated value of the indicator.

† Ultra-processed foods: sweet or savoury biscuits/cookies; baby cereals; yogurts; carbonated drinks; other sweetened beverages (packed juice, packed coconut water, guarana syrups, redcurrant syrup, powdered juice or fruit juice with added sugar); sweets and treats; sausages and processed meats; and packaged snacks, packaged bread and instant noodles. There is an overlap of foods from the ultra-processed group with the groups ‘grains, roots, tubers and plantains’, ‘dairy products’ and ‘flesh foods’.

Among children living in severe food poverty, the most reported food group combinations were ‘breast milk only’ (10·1 % (95 % CI 5·9, 14·3)), ‘breast milk and/or dairy products’ (12·3 % (95 % CI 8·1, 16·4)), ‘and breast milk and/or dairy products’ + ‘grains, roots, tubers, and plantains’ (32·1 % (95 % CI 25·5, 38·8)). The less frequent combinations were aggregated as ‘other combinations of food groups’, which included (i) ‘breast milk and/or dairy products’ + ‘legumes’; ‘breast milk and/or dairy products’ + ‘flesh foods’, (ii) ‘breast milk and/or dairy products’ + ‘eggs’, (iii) ‘breast milk and/or dairy products’ + ‘vitamin A-rich fruits and vegetables’, (iv) ‘breast milk and/or dairy products’ + ‘other fruits and vegetables’ and (v) other combinations of food groups excluding breast milk and dairy products (Supplementary Table 2).

**Fig. 1:**
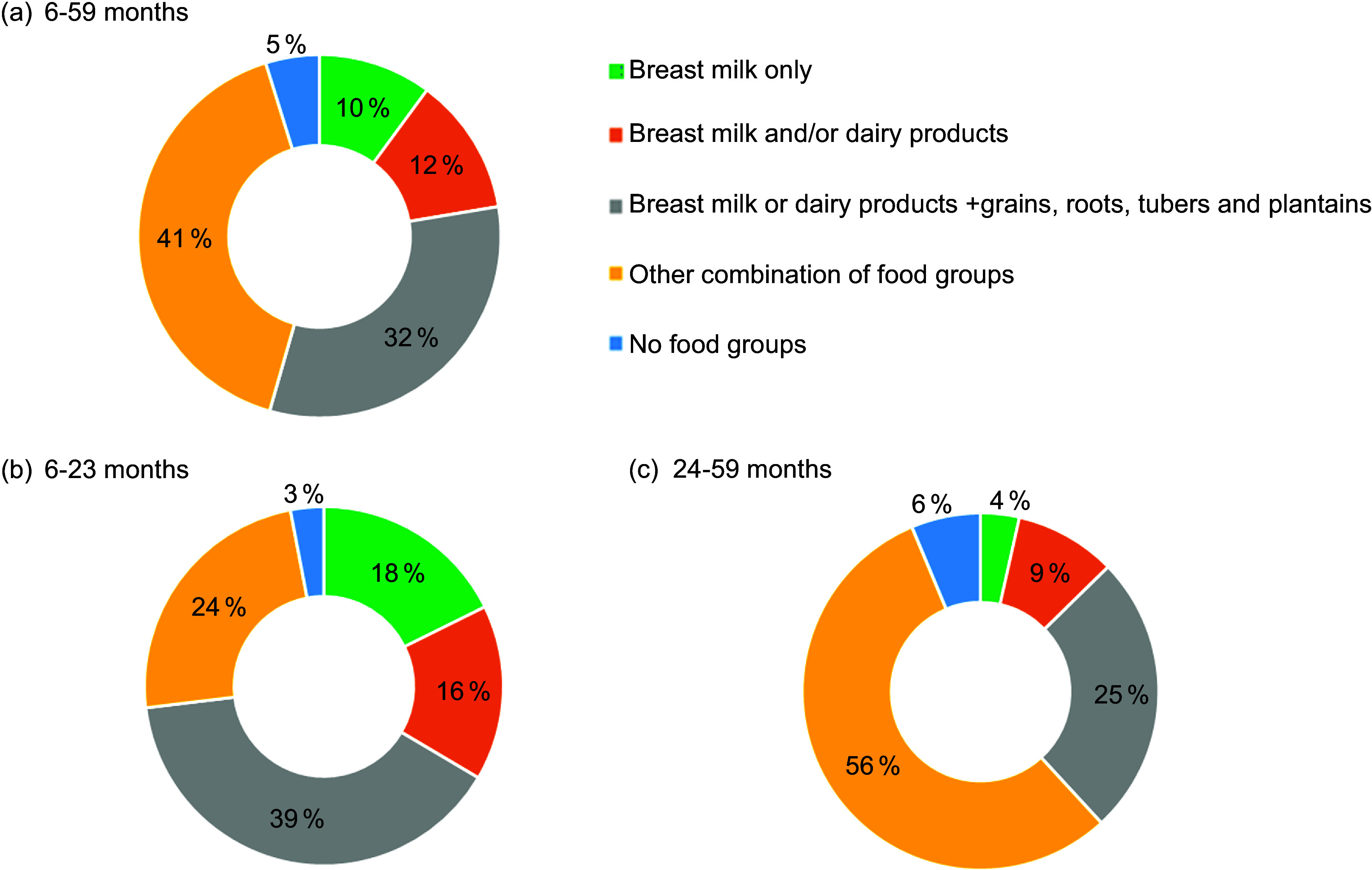
Prevalence of consumption of different combinations of food groups in children living in severe food poverty according to age. ENANI-2019 Note: No food groups: Children consumed only ultra-processed foods that are not included in any of the eight food groups or children who did not eat any listed food in the day before the interview or children who did not eat any food.

**Fig. 2:**
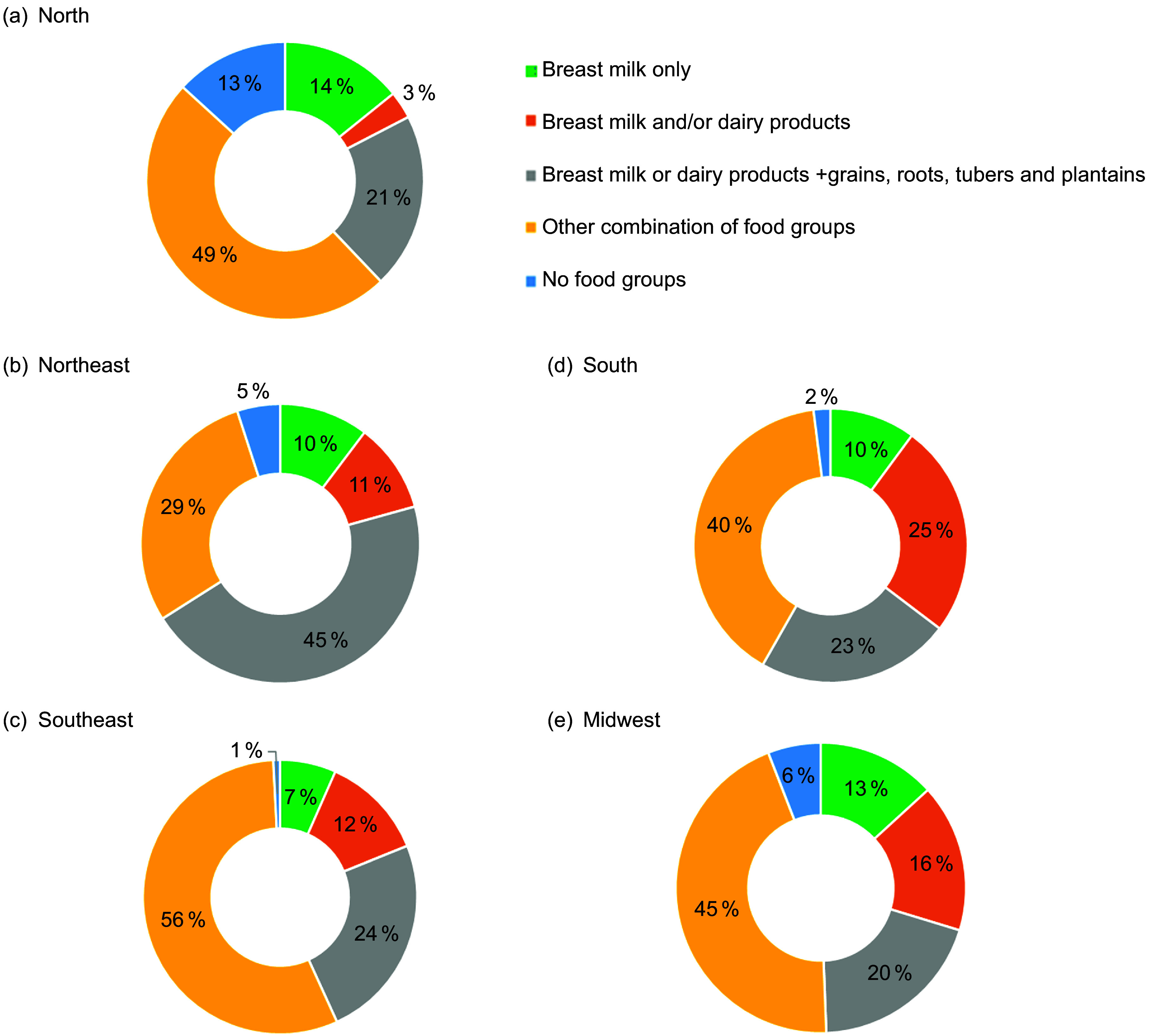
Prevalence of consumption of different combinations of food groups in children aged 6–59 months living in severe food poverty according to macro-regions. ENANI-2019 Note: No food groups: Children consumed only ultra-processed foods that are not included in any of the eight food groups or children who did not eat any listed food in the day before the interview or children who did not eat any food.

The combination of ‘breast milk and/or dairy products’ plus ‘grains, roots, tubers, or plantains’ was the most frequent one in all age groups. ‘Breast milk only’ was consumed by 10 %, 18 % and 4 % of children aged 6–59, 6–23 and 24–59 months, respectively (Fig. 1). The prevalence of ‘breast milk only’ consumption varied from 7 to 14 % across the geographic regions, being higher in the North. ‘Breast milk and/or dairy products’ plus ‘grains, roots, tubers, or plantains’ was the most frequent combination in all geographic regions except the South (Fig. 2).

The prevalence of food combinations consumed by children suffering from severe food poverty was comparable across Brazil’s geographic regions, except for the North and Northeast regions. Milk, cereals and tubers were widely consumed in the Northeast, while the percentage of children exclusively consuming milk (breast milk and cow milk products) was lower in the North (Fig. 2). The less frequent combinations consumed by children aged 6–59 months living in severe food poverty were ‘breast milk and/or dairy products’ plus ‘legumes’ and ‘breast milk and/or dairy products’ plus ‘vitamin A-rich fruits and vegetables’, that is, 0·6 % and 0·3 % of the children, respectively. Consumption of only UPF was observed in 2 % of children living in severe food poverty (Supplementary Table 2).

**Fig. 3:**
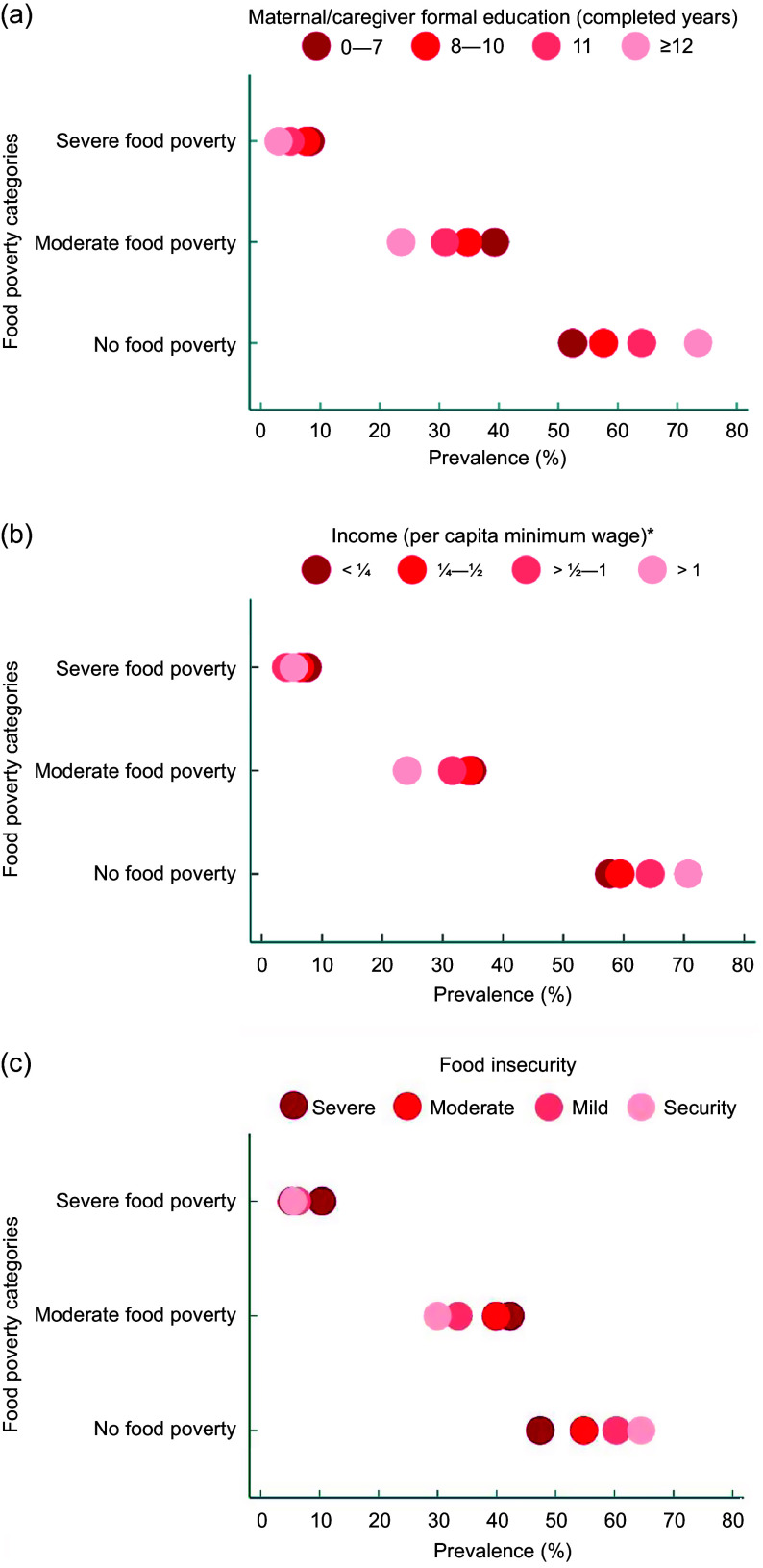
Prevalence of children aged 6–59 months living in food poverty categories according to mother/caregiver level of education, income and food insecurity level. ENANI-2019 Note: *Minimum wage for 2019 = R$ 998·00 (∼ US$ 252·88) and for 2020 = R$ 1,039·00 (∼ US$ 247·90).

The lowest maternal/caregiver level of education and income categories showed the highest prevalence of children experiencing severe and moderate food poverty. Greater inequality between levels of education was observed for the moderate and no food poverty categories (Fig. 3). The highest prevalence of children living in severe and moderate food poverty was also observed in the most vulnerable socio-economic categories of mother/caregiver level of education, income and to a less extent for food insecurity results that were not statistically significant (Fig. 3 and Supplementary Table 3).

## Discussion

This study describes for the first time an overview of food poverty in Brazilian children aged 6–59 months based on data from a national survey representing 15 million infants. In 2019, 38·5 % of these children lived in food poverty (6 % severe and 32·5 % moderate). Severe food poverty was more frequent among children aged < 2 years and those living in the Northeast region. The prevalence of severe food poverty was higher in children whose mothers/caregivers had lower levels of education (< 8 years) compared with those with higher education (11 years and ≥12) and those from low-income families (per capita minimum wage < ¼ compared with > ½–1). Notably, the food groups most consumed at all ages were grains, roots, tubers, plantains, flesh foods and dairy products. The study also highlights the alarming fact that 64·6 % of children living in severe food poverty consumed UPF products.

Even though the results are concerning, the prevalence of food poverty in Brazil in 2019 was lower than the global average. UNICEF’s 2022 report shows that globally 71 % of children aged < 5 years are living in food poverty, with 30 % in the severe form. In Ethiopia, Somalia, South Sudan, Chad and Afghanistan, > 35 % of the children live in severe food poverty. In Latin American and Caribbean nations, the prevalence of severe and moderate food poverty is 10 % and 28 %, respectively, similar to those reported in Brazil^(12)^.

It is well established that children aged < 5 years consume UPF frequently^(17,25)^. In the present study, even those who lived in severe food poverty had substantial access to this food group. Regular consumption of UPF can worsen nutritional deficiencies caused by severe food poverty and harm children’s health^(19,26,27)^. In another study with children aged 6–23 months from the ENANI-2019, those with white skin colour/race and higher maternal/caregiver level of education had a higher prevalence of consuming an appropriate diet (meeting the MDD without UPF)^(17)^. The tools used to evaluate the nutritional value of children’s diets must detect UPF intake. There was a slight increase in UPF consumption in Brazil between 2008/2009 and 2017/2018 among the general population over 10 years old (1·02 percentage points), which was more substantial in the first-income quintile (3·54 percentage points). The decline in UPF prices since the early 2000s, increased supermarket sales and targeted marketing to the most vulnerable individuals have contributed to this trend^(28)^.

On the other hand, it is worth considering the design and implementation of structural measures within policies and programmes to promote healthier expenditure of money used for foods. In line with this, the Brazilian government introduced new nutritional labelling in 2020, highlighting the presence of significant amounts of added sugar, saturated fat and sodium^(29)^. Tax reform was also approved in 2023, including the taxation of products and services that are harmful to health or the environment. The inclusion of UPF in the list of harmful products is being debated. Moreover, this reform also approved the removal of taxes on items in the basic food basket, which should consist of healthy foods^(30)^. Implementing these measures is expected to reduce the consumption of UPF. Food poverty is a significant concern in the North and Northeast geographic regions, affecting 47 % of children. This alarming data is consistent with the poorest socio-economic indicators^(31)^, restricted access to healthcare services^(32,33)^ and higher prevalence of severe food insecurity (10 % and 7 % in 2017/2018, respectively) in these geographic regions^(31)^. A previous study has shown that children living in the Northeast region of Brazil are less likely to have MDD than a children living in other areas of the country^(34)^. The lack of MDD is more prevalent among children from low-income families or those with less educated mothers^(10,34,35)^, conditions that increase the social vulnerability of families and children^(12)^.

Inequalities in health within the maternal and child populations are prevalent across different countries and within subgroups of the same nation. Substantial disparities were identified in the access to antenatal care, the prevalence of stunting, < 5 years mortality rate and care-seeking for children with pneumonia symptoms, among others^(36)^. This study observed that children from low-income families and those with lower maternal/caregiver education levels have a higher prevalence of moderate and severe food poverty. It is possible to visualise a noticeable gradient in the prevalence according to these categories within equiplots. The opposite was observed among children who achieved MDD. The connection between low income, low educational levels and food poverty is unsurprising. Still, it was revealed to be a pressing issue, highlighting the importance of implementing social initiatives that ensure educational and income opportunities for the most disadvantaged communities.

Children living in severe food poverty in Brazil have a different food profile than the global average^(12)^. However, the top three most consumed food groups remain breast milk, breast milk combined with cow’s milk or dairy products, and milk combined with cereals or tubers (energy-dense food groups but low in micronutrient content). In Brazil, 54 % of children living in severe food poverty consume these groups of foods, compared with 90 % globally^(12)^. No matter what combination of food groups these children consume, their diets will always lack the necessary nutrients. The unexpected differences in the intake between the North and Northeast geographic regions are worth considering. The consumption of solely breast milk accounted for 13 % and 5 %, while the combination of ‘breast milk and/or dairy products’ and ‘grains, roots, tubers and plantains’ represented 21 % and 45 %, respectively. It is necessary to explore these data further to understand whether these differences are cultural or related to the local availability of foods.

The outcomes of failing to attain MDD during infancy and early childhood are linked to growth and developmental deficiencies. Cohort studies on this age group revealed that low dietary diversity is associated with growth deficit^(37)^ and low child development scores^(37,38)^ compared with children who achieved MDD. Therefore, actions and strategies to promote and encourage dietary diversity for children aged < 2 years should be of concern.

The present study offers a remarkable contribution by presenting the food poverty indicator in Brazil for the first time, which has been used on a large sample of households with children aged 6–59 months. This indicator can help to devise novel health and nutrition strategies to address the needs of Brazilian children better.

The study has some limitations related to the questions in the structured questionnaire. There was a question about fruit intake and one about fruit sources of vitamin A. If the mother/caregiver said yes to both questions and the child only ate fruit with vitamin A, it would appear that they consumed two of eight food groups. This may overestimate fruit consumption and underestimate food poverty. The absence of a question related explicitly to cheese intake may lead to an underestimation of dairy consumption and an overestimation of food poverty. Still, this effect tends to be negligible, given the low consumption of this food among the population being studied. Another constraint is inherent to the questionnaire format, as the child might have eaten certain foods that were not inquired. Nevertheless, the survey was conducted by specialists from various geographic regions of Brazil to create a comprehensive questionnaire that would cover all food groups that contribute to the food poverty indicator.

Food poverty was highly prevalent among Brazilian children aged 6–59 months, whose diet relied on milk and grain consumption and UPF. The limited options available for the children restrict the nutritional value of their diet. This scenario may have worsened with the health and social consequences of the COVID-19 pandemic and increased food insecurity in Brazilian households with children aged < 10 years^(39)^. Despite the challenges faced, there are positive signs towards improving the quality of life of the Brazilian population. Promising initiatives include expanding the family health strategy^(40)^ and reintroducing government policies to safeguard citizens’ rights after the 2022 presidential elections (including improvements to the conditional income transfer programme). These efforts can potentially bring positive changes for children aged < 5 years. Governments can address the underlying issues behind food poverty by implementing effective intersectoral policies and programmes focusing on poverty, unequal resource distribution and lack of education.

## Supporting information

Carneiro et al. supplementary material Carneiro et al. supplementary material

## Supplementary material

For supplementary material accompanying this paper visit https://doi.org/10.1017/S1368980024001435
