# Supplementary material for: Food poverty among children aged 6–59 months in Brazil: results from the Brazilian National Survey on Child Nutrition (ENANI-2019)
Source: Public Health Nutr. 2024 Sep 13;27(1):e162. doi: 10.1017/S1368980024001435 (PMC11504526; doi:10.1017/S1368980024001435)
Supplement: Carneiro et al. supplementary material [file S1368980024001435sup001.docx]

**Supplementary Table 1** - Relative frequency of food groups consumed by children aged 6–59 months according to age. ENANI-2019.

| **Food groups by age** | Relative frequency | |
| --- | --- | --- |
| **6-59 months** | % | 95% CI |
| Breast milk | 24.3 | 22.8; 25.7 |
| Grains, roots, tubers and plantains | 94.3 | 93.5; 95.1 |
| Legumes | 71.3 | 68.8; 73.8 |
| Dairy products | 85.0 | 83.4; 86.7 |
| Flesh foods | 81.6 | 79.7; 83.4 |
| Eggs | 20.0 | 18.5; 21.6 |
| Vitamin A-rich fruits and vegetables | 36.8 | 34.5; 39.2 |
| Other fruits and vegetables | 65.4 | 62.6; 68.2 |
| Ultra-processed foods | 88.8 | 87.0; 90.7 |
| **6-23 months** |  |  |
| Breast milk | 51.4 | 47.9; 55.0 |
| Grains, roots, tubers and plantains | 90.2 | 88.5; 91.9 |
| Legumes | 64.8 | 61.6; 68.0 |
| Dairy products | 81.0 | 78.4; 83.7 |
| Flesh foods | 70.4 | 67.4; 73.4 |
| Eggs | 14.0 | 12.1; 16.0 |
| Vitamin A-rich fruits and vegetables | 43.2 | 39.7; 46.7 |
| Other fruits and vegetables | 68.6 | 66.1; 71.1 |
| Ultra-processed foods | 80.5 | 77.2; 83.8 |
| **24-59 months** |  |  |
| Breast milk | 10.6 | 9.3; 12.0 |
| Grains, roots, tubers and plantains | 96.3 | 95.5; 97.2 |
| Legumes | 74.6 | 72.0; 77.3 |
| Dairy products | 87.0 | 85.2; 88.9 |
| Flesh foods | 87.2 | 85.2; 89.3 |
| Eggs | 23.0 | 21.0; 25.1 |
| Vitamin A-rich fruits and vegetables | 33.6 | 31.2; 36.0 |
| Other fruits and vegetables | 63.8 | 60.2; 67.4 |
| Ultra-processed foods | 93.0 | 91.4; 94.6 |

Note: Details of the eight food groups included in the UNICEF indicator of food poverty: 1) breast milk; 2) grains, roots, and tubers such as porridge, bread, rice, pasta, potatoes, and other starchy vegetables; 3) legumes including beans, lentils, peas, and chickpeas; 4) milk and dairy products like infant formula, animal milk, and yogurt; 5) flesh foods that contain animal meat, liver, kidney, heart, sausages, and processed meats; 6) eggs; 7) vitamin A-rich fruits and vegetables such as carrot, pumpkin, sweet potato, cabbage, spinach, and other local dark green leafy vegetables; and 8) other fruits and vegetables. Ultra-processed foods: sweet or savory biscuits/cookies; baby cereals; yogurts; carbonated drinks; other sweetened beverages (packed juice, packed coconut water, guarana syrups, redcurrant syrup, powdered juice, or fruit juice with added sugar); sweets and treats; sausages and processed meats; packaged snacks, packaged bread, and instant noodles.

**Supplementary Table 2** - Prevalence of different food combinations in children aged 6–59 months in severe food poverty. ENANI-2019.

| **Combinations food groups** | **Prevalence** | |
| --- | --- | --- |
|  | **%** | **95% CI** |
| Breast milk only | 10.1 | 5.9; 14.3 |
| Breast milk and/or dairy products | 12.3 | 8.1; 16.4 |
| Breast milk and/or dairy products + grains, roots, tubers and plantains | 32.1 | 25.5; 38.8 |
| Breast milk and/or dairy products + legumes | 0.6 | 0.0; 1.2 |
| Breast milk and/or dairy products + flesh foods | 5.3 | 2.3; 8.4 |
| Breast milk and/or dairy products + eggs | 1.6 | 0.0; 3.6 |
| Breast milk and/or dairy products + vitamin a-rich fruits and vegetables | 0.3 | 0.0; 0.7 |
| Breast milk and/or dairy products + other fruits and vegetables | 6.5 | 3.3; 9.7 |
| Other combination of food groups excluding breast milk and dairy products | 26.4 | 21.0; 31.9 |
| Ultra-processed foods only | 2.2 | 0.0; 4.7 |
| None of the food groups listed | 2.6 | 0.0; 6.5 |

Note: severe food poverty: consumption of 0–2 food groups of the eight food groups (i.e. 1) breast milk; 2) grains, roots, and tubers such as porridge, bread, rice, pasta, potatoes, and other starchy vegetables; 3) legumes including beans, lentils, peas, and chickpeas; 4) milk and dairy products like infant formula, animal milk, and yogurt; 5) flesh foods that contain animal meat, liver, kidney, heart, sausages, and processed meats; 6) eggs; 7) vitamin A-rich fruits and vegetables such as carrot, pumpkin, sweet potato, cabbage, spinach, and other local dark green leafy vegetables; and 8) other fruits and vegetables).^a^Ultra-processed foods: sweet or savory biscuits/cookies; baby cereals; yogurts; carbonated drinks; other sweetened beverages (packed juice, packed coconut water, guarana syrups, redcurrant syrup, powdered juice, or fruit juice with added sugar); sweets and treats; sausages and processed meats; packaged snacks, packaged bread, and instant noodles.

**Supplementary Table 3** - Prevalence of food poverty and no food poverty in Brazilian children aged 6–59 months according to socioeconomic characteristics. ENANI-2019.

| **Variables** | **Severe food poverty** | | **Moderate food poverty** | | **No food poverty** | |
| --- | --- | --- | --- | --- | --- | --- |
|  | **%** | **95% CI** | **%** | **95% CI** | **%** | **95% CI** |
| **Brazil** | 6.0 | 5.0; 6.9 | 32.5 | 30.1; 34.9 | 61.6 | 58.7; 64.4 |
| **Mother/caregiver formal education (completed years)** | | | | | | |
| 0–7 | 8.3 | 6.2; 10.4^a,b^ | 39.3 | 36.1; 42.6^a,b^ | 52.4 | 48.3; 56.4 |
| 8–10 | 7.6 | 5.7; 9.6 | 34.8 | 30.3; 39.2 | 57.6 | 52.8; 62.4 |
| 11 | 5.0 | 3.8; 6.1^a^ | 31.0 | 28.0; 34.1^a^ | 64.0 | 60.5; 67.5 |
| ≥12 | 3.0 | 1.8; 4.2^b^ | 23.6 | 19.5; 27.6^b^ | 73.5 | 69.0; 77.9 |
| **Income (*per capita* minimum wage)** | | | | | | |
| < ¼ | 7.5 | 5.6; 9.4^c^ | 34.8 | 31.3; 38.2 | 57.7 | 53.6; 61.8 |
| ¼–½ | 6.3 | 4.5; 8.1 | 34.3 | 30.9; 37.8 | 59.4 | 55.2; 63.6 |
| > ½–1 | 4.1 | 2.9; 5.3^c^ | 31.6 | 28.1; 35.0 | 64.4 | 60.7; 68.1 |
| > 1 | 5.3 | 3.1; 7.4 | 24.1 | 19.2; 28.9 | 70.7 | 65.1; 76.3 |
| **Food insecurity** | | | | | | |
| Severe insecurity | 10.4 | 5.5; 15.2 | 42.3 | 32.8; 51.7 | 47.4 | 36.6; 58.1 |
| Moderate insecurity | 5.3* | 2.0; 8.5 | 39.9 | 32.8; 47.0 | 54.8 | 47.8; 61.8 |
| Mild insecurity | 6.2 | 4.4; 7.9 | 33.5 | 29.9; 37.1 | 60.3 | 55.6; 65.1 |
| Security | 5.5 | 4.4; 6.7 | 30.0 | 26.7; 33.3 | 64.5 | 60.6; 68.4 |
| **Skin color/ race** | | | | | | |
| White | 5.9 | 4.4; 7.4 | 30.5 | 27.3; 33.6 | 63.6 | 59.9; 67.4 |
| Brown | 6.2 | 4.8; 7.6 | 33.6 | 30.7; 36.5 | 60.3 | 56.9; 63.6 |
| Black | 5.0 | 2.5; 7.4 | 35.7 | 29.6; 41.9 | 59.3 | 52.7; 65.9 |

Note: severe food poverty: consumption of 0–2 food groups; moderate poverty: consumption of 2–4 food groups; and no food poverty (i.e. with minimal dietary diversity): consumption of five or more of the eight food groups (i.e. 1) breast milk; 2) grains, roots, and tubers such as porridge, bread, rice, pasta, potatoes, and other starchy vegetables; 3) legumes including beans, lentils, peas, and chickpeas; 4) milk and dairy products like infant formula, animal milk, and yogurt; 5) flesh foods that contain animal meat, liver, kidney, heart, sausages, and processed meats; 6) eggs; 7) vitamin A-rich fruits and vegetables such as carrot, pumpkin, sweet potato, cabbage, spinach, and other local dark green leafy vegetables; and 8) other fruits and vegetables). ^a^ Significant difference between 0–7 and 11 years education level; ^b^ significant difference between 0–7 and ≥12 years education level; ^c^ significant difference between income (*per capita* minimum wage) < ¼ and > ½–1. *Coefficient of variation (CV) ≥ 30%. CV is a measure of dispersion that indicates data heterogeneity, obtained by the ratio between the standard error and the estimated value of the indicator.
